# Methods for motion artifact reduction in online brain-computer interface experiments: a systematic review

**DOI:** 10.3389/fnhum.2023.1251690

**Published:** 2023-10-18

**Authors:** Mathias Schmoigl-Tonis, Christoph Schranz, Gernot R. Müller-Putz

**Affiliations:** ^1^Laboratory of Collaborative Robotics, Department of Human Motion Analytics, Salzburg Research GmbH, Salzburg, Austria; ^2^Institute of Neural Engineering, Laboratory of Brain-Computer Interfaces, Graz University of Technology, Graz, Austria; ^3^BioTechMed Graz, Graz, Austria

**Keywords:** brain-computer interface (BCI), electroencephalography (EEG), artifact removal, motion artifact, muscle artifact, fasciculation, cable swing

## Abstract

Brain-computer interfaces (BCIs) have emerged as a promising technology for enhancing communication between the human brain and external devices. Electroencephalography (EEG) is particularly promising in this regard because it has high temporal resolution and can be easily worn on the head in everyday life. However, motion artifacts caused by muscle activity, fasciculation, cable swings, or magnetic induction pose significant challenges in real-world BCI applications. In this paper, we present a systematic review of methods for motion artifact reduction in online BCI experiments. Using the PRISMA filter method, we conducted a comprehensive literature search on PubMed, focusing on open access publications from 1966 to 2022. We evaluated 2,333 publications based on predefined filtering rules to identify existing methods and pipelines for motion artifact reduction in EEG data. We present a lookup table of all papers that passed the defined filters, all used methods, and pipelines and compare their overall performance and suitability for online BCI experiments. We summarize suitable methods, algorithms, and concepts for motion artifact reduction in online BCI applications, highlight potential research gaps, and discuss existing community consensus. This review aims to provide a comprehensive overview of the current state of the field and guide researchers in selecting appropriate methods for motion artifact reduction in online BCI experiments.

## Introduction

Non-invasive brain-computer interface (BCI) research based on electroencephalography (EEG) has a long scientific history (e.g., Vidal, 1973; Sherman et al., 1984; Wolpaw et al., 2002; Neuper et al., 2006; Sejnowski et al., 2007; Käbler et al., 2014, to name a view), but only in recent years research projects started to investigate the effects of more excessive forms of motion artifacts in EEG, caused by simultaneous execution of disruptive motion tasks like treadmill walking or passive induction (e.g., Scherer et al., 2014; Seeber et al., 2014; Wagner et al., 2014; He et al., 2018; Vidaurre et al., 2021, …).

###  Brain-computer interfaces

Brain-computer interfaces enable a direct pathway for communication between the brain and a technical device. They allow a user to actively send commands by analyzing complex signals from detectable human brain patterns. Invasive methods require surgery so that electrodes can either be implanted on the brain tissue (usually subdurally) or intracortically (highly invasive). Invasive methods are primarily applied in the clinical domain e.g., in patients with limited or no movement or communication capabilities. Non-invasive BCIs measure the brain activity from outside the head and can be worn as caps, headsets, helmets or other wearables. Non-invasive BCIs based on EEG are expected to make up the biggest proportion of the future market as only EEG can be applied easily on the intact head to be used in less static scenarios. While invasive and non-invasive BCIs are already applied as health devices for therapy in e.g., rehabilitation centers or as communication tools for people with e.g., spinal cord injury, there are other application domains where its potential is not yet fully exploited (e.g., sports with wearables, collaborative industry with co-working robots, more dynamic rehabilitation exercise therapies, the gaming industry and several more). One major reason for this is a high amount of recorded background noise (artifacts) due to other activities being executed simultaneously to the neural command interpretation, which leads to a poor overall signal-to-noise ratio (SNR).

###  Objectives and research question

In this systematic literature review we publish a comprehensive table of devices, software tools, methods and algorithms to correct, reduce, remove or mitigate artifacts caused by motion originating from muscle activity, fasciculation, cable swings or other whole body motion effects in the human EEG data. Moreover, through paper-wise comparisons of different processing pipelines and similarities between pipelines of different authors, we conclude additional insights in EEG analysis. Furthermore, potential research gaps and community consensus in all investigated literature are presented. In order to be able to investigate the domain in the lab, first we tried to research all relevant existing methods and systems publicly listed on Pubmed. We objectively compared sensor types, system setups, processing pipelines, software toolkits, mathematical methods and algorithms for application in a real-world scenario (noise coming from e.g., standing, walking, collaborative work, …).

###  Other existing systematic reviews

During our search we found existing reviews with different research questions. We excluded them from the reviewed literature as we wanted to create a lookup table of existing methods originating from the literature where they were originally first introduced to the community. In demarcation to existing reviews we present a short comparison table of all dismissed literature reviews (Table 1).

**Table 1:** List of found and reviewed other existing systematic reviews covering closely related topics.

**Publication title**	**Literature review goals**
Upper-Body Post-activation Performance Enhancement for Athletic Performance: A Systematic Review with Meta-analysis and Recommendations for Future Research (Finlay, 2022)	Literature Research on post-activation performance enhancement (PAPE) in upper-body movement.
Analysis of Human Gait Using Hybrid EEG-fNIRS-Based BCI System: A Review (Khan, 2021)	Literature Research on commonly used signal processing and machine learning algorithms on human gait analysis for hybrid BCIs.
Applications of EEG indices for the quantification of human cognitive performance: A systematic review and bibliometric analysis (Ismail, 2020)	Literature Research on the applications of EEG indices for quantifying human performance in a variety of cognitive tasks (macro and micro scales).
Technological advancements and opportunities in Neuromarketing: a systematic review (Rawnaque, 2020)	Literature Research on technological advancements in Neuromarketing over previous 5 years in selected 57 papers.
The Potential of Functional Near-Infrared Spectroscopy-Based Neurofeedback—A Systematic Review and Recommendations for Best Practice (Kohl, 2020)	Literature Research on fNIRS neurofeedback studies with a focus on training protocols, online signal-processing methods and evaluation of quality and effectiveness.
Neuroimaging of Human Balance Control: A Systematic Review (Wittenberg, 2017)	Literature Research on neural correlates underlying static and dynamic human balance control, with aims to support future mobile neuroimaging research.
State-of-the-Art Analysis of High-Frequency (Gamma Range) Electroencephalography in Humans (Nottage, 2015)	Literature Research on gamma oscillations, focus on recent progress made for artifacts in power line, saccade-associated extra-ocular muscle contraction and blinking, activity of muscles of scalp, face and neck and screen refresh artifacts.
High-frequency brain activity and muscle artifacts in MEG/EEG: a review and recommendations (Muthukumaraswamy, 2013)	Literature Research on the spectral, spatial, and temporal characteristics of muscle artifacts are compared for high-frequency neural activity. Several developed techniques to help suppress muscle artifacts in MEG/EEG are reviewed.
Mapping Hemodynamic Correlates of Seizures Using FMRI: A Review (Chaudhary, 2011)	Literature Research on the various fMRI-EEG acquisition and data analysis methods applied to map epileptic seizure-related hemodynamic changes.
Brain Computer Interfaces, a Review (Nicolas-Alonso, 2012)	Literature Research on the standard BCI: signal acquisition, preprocessing or signal enhancement, feature extraction, classification and control interface.
The use of electroencephalography in language production research: a review (Ganushchak, 2011)	Literature Research on available results of overt speech production involving EEG measurements, such as picture naming, Stroop naming, and reading aloud.
Electromyogenic Artifacts and Electroencephalographic Inferences (Shackman, 2009)	Literature Research on intra-individual GLM-based methods to correct artifacts in EMG and EEG.

From the table above it is possible to derive that there have not yet been attempts to create a full comprehensive lookup table containing all used methods that process body motion artifacts in EEG recordings. Some of the found literature reviews had a much more specialized focus trying to demonstrate individual methods for a specific target domain or a more specific research problem (Shackman, 2009; Muthukumaraswamy, 2013; Nottage, 2015; Kohl, 2020; Khan, 2021), while others had a more broad coverage of BCIs in general (Nicolas-Alonso, 2012). Other found reviews had muscle activation and body motion as a focus but did not attempt to list methods for artifact suppression (Wittenberg, 2017; Finlay, 2022). Chaudhary (2011); Ganushchak (2011); Ismail (2020) and Rawnaque (2020) had a focus on entirely different topics, but still showed up in the literature search as they had all required keywords we searched for.

## Methods

To conduct this study we used the PRISMA method (http://www.prisma-statement.org/) to find publications of interest in a very large pool of search results. This means we first defined search terms and a filtering rule set and then applied our definitions to the found results to reduce the number of papers included in the review.

###  Study design

As starting point, we selected the literature database and defined the search terms and conditions, and finally we defined filter criteria to select papers to be included into the review.

###  Search strategy

The basis for our search was the Pubmed database (https://pubmed.ncbi.nlm.nih.gov/). Into the search we included all papers published until May, 31st 2022 and which were publicly available. The following search terms were defined:

- Term A) “EEG Muscle (Artifact OR Artifact)” (390 search results)
- Term B) “EEG Motion (Artifact OR Artifact)” (236 search results)
- Term C) “EEG (Artifact OR Artifact) Reduction” (309 search results)
- Term D) “EEG (Artifact OR Artifact) Removal” (918 search results)
- Term E) “EEG (Artifact OR Artifact) Rejection” (278 search results)
- Term F) “EEG (Artifact OR Artifact) Mitigation” (20 search results)
- Term G) “EEG (Artifact OR Artifact) Detection” (939 search results)
- Term H) “Cable Motion (Artifact OR Artifact)” (36 search results)
- Term I) “Cable Swing” (40 search results)
- Term J) “Electrode pops” (44 search results).

All search terms result in a total number of 3,210 publications. We removed duplicate results by combining all search terms with a logical OR and the final result was 2,333 publications. Additionally, we applied the existing search filters “Abstract” and “Free full text” of the Pubmed search interface, which left us with 747 search results total. Yet, even of those 747 publications there still were 40 papers non accessible for download or full text view anyways, due to non-available external server links or other system failures during download time. We continued with our defined rule set with the remaining unique 707 open access publications. We will discuss this in detail in the next sections.

###  Filter criteria

We manually evaluated the 707 found literature results by applying the following filter rules:

- ***NO_OPEN_ACCESS***: do exclude every search result where there is no free downloadable or web viewable full text available (“we only consider publicly available full text publications—OPEN ACCESS”).
- ***IS_SYSTEMATIC_REVIEW***: do exclude all other systematic reviews (“we want to evaluate only original methods published in the individual papers were they have been introduced to the community”).
- ***NO_NI_HUMAN_EEG***: do exclude all studies using animals, studying animal brains, using invasive BCI systems, using non-EEG based BCI systems (“research is based on non-invasive human EEG”).
- ***NO_ARTIFACT_FOCUS***: do exclude all studies with non-technical focus; exclude all studies whos main result is not interested in artifact reduction/removal (“research is primarily interested in demonstrating technical methods to filter EEG motion artifacts”).
- *NO_MOTION_FOCUS*: do exclude all the other artifact sources like e.g., eye saccades, eye blink, heartbeat, non-physiological sources, … (“research focuses on motion artifacts originating from muscle artifacts, fasciculation, artifacts through body motion, or cable swings”).
- ***NO_REAL_DATASET***: do exclude all theoretical studies without real participants data (“research needs to have conducted online or offline studies and created data with real participants or re-used existing data recorded from real participants”).
- ***NO_SCIENCE_GRADE***: do exclude all studies that did not use science graded EEG devices (“research needs to have used science graded EEG systems in their studies”).

These filter rules have been manually applied in the order given above for every found search result. It should be noted that many papers failed the above criteria for multiple rules at once. We also found multiple papers that would partially fulfill filter criteria e.g., through achieving more than one goal in the presented work. In case of an uncertain fit we made the decision to remove the work. A total number of 77 papers passed the presented filter rules and were added to the final pipeline lookup table (detailed information will follow in Result section).

###  Information extraction

All extracted information of all papers can be found online in the corresponding Github repository under https://github.com/iot-salzburg/SLR_on_motion_artifact_reduction_for_BCI. The main file which summarizes all extracted information is called “Systematic Literature Review on Motion Artifact Removal of EEG Signals.xlsx”. In this paper we refer to this document as “the lookup table”. In this section we describe the extracted information of every paper and where to find it within the lookup table.

From each paper, we extracted the following information: (i) the paper's objective, (ii) the data acquisition, (iii) the number of participants and demographics, (iv) the mental strategy utilized (e.g., “brain-teaser tasks,” “motor imagery tasks,” “non-motor imagery tasks,” “dynamic visualization tasks,” “attention strategy,” “motor execution”), (v) the evaluation metrics, (vi) the software framework for building the pipeline (“Matlab,” “Python,” or “unknown”), (vii) code availability, (viii) the main innovation, (ix) and findings and further work suggested. This information can be found in the “papers_annotated” tab of the lookup table.

For every paper, the pipelines with all pipeline components and their results were added to the final lookup table into separate table tabs: The “pipeline” table specifies the pipelines used in each paper, which is a combination of various methods including data preprocessing and artifact detection algorithms, applied to the raw data to get a cleaned EEG signal or perform a task.

The “result” table presents the comparisons of the pipelines per paper for a specific setup, data, and evaluation metric using a rank-based approach. With this rank-based approach we tried to show the comparison results of the original authors themselves. The ranking shown here, therefore is simply the ranking the original authors assigned to their pipelines using the performance metric of their choice (e.g., SNR, sensitivity, specificity, classification accuracy, correlation coefficients, ERD peak scores, f-scores, visual inspection, …). In cases where ties occurred, ranks were assigned evenly to maintain consistency in the result analysis. For example, if four pipelines were compared and one was significantly the best, two were tied for second place, and one was significantly the worst, the ranks assigned would be “1,” “2.5,” “2.5,” and “4.” This ensured that the median pipelines had the same distance to the best and worst pipelines for further analysis of the results.

In the tables “devices,” “software,” “motion artifact removal methods,” and “classification models” all unique approaches are summarized and semantically grouped. Note that it was only possible to add any information here, if it was clearly declared by the paper authors within the paper itself.

In the “consensus”-table, common understanding of the investigated papers is presented and similarities of statements made across authors are being summarized. For the “research gap”-table individual suggested work or potential research gaps are listed. This involves found research gaps by paper authors, as well as potential research gaps assumed by the authors of this systematic literature review.

**Figure 1:**
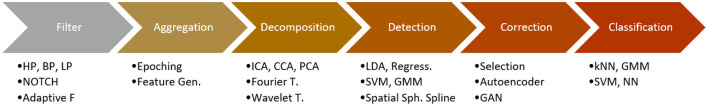
Generic pipeline with exemplary methods for each category.

In Figure 1, an overview of a generic pipeline for motion artifact detection, correction, reduction, or removal in EEG data is presented:

The pipeline is designed to process raw EEG data and produce artifact-detected, corrected, reduced, or removed EEG data as output. The methods used within the pipeline are categorized into six main categories: (1) filters, (2) aggregations such as epoching and feature generation, (3) decomposition methods such as blind source separation, (4) artifact detection without correction, (5) artifact correction methods, and (6) classification models used for the actual BCI task also known as downstream task. Additionally, some pipelines contain specialized methods that are only used in BCI experiments or they reuse previously introduced subpipelines that don't fit any specific category. We grouped these cases into the category “Special algorithms”.

The order of the methods within the pipeline is specified to allow more detailed investigations. However, it should be noted that the order-based approach is not always able to exactly determine the pipeline, as some pipelines may involve parallel streams, iterations, and recursions, and moreover might differ in their parameters. Due to the lack of detailed information in some papers regarding their pipeline's architecture and implementation, a more precise pipeline modeling on the same level of granularity was not possible.

###  Comparison of pipelines

As mentioned, a pipeline is represented here by an ordered application of filters, decomposition methods, artifact detection algorithms, and other methods on the raw contaminated EEG data. This method composition of a given pipeline, as well as the setup of the experiment in each paper, may vary strongly from other pipelines presented in the list. The presented pipelines are evaluated and compared by three criteria, which we defined: “online score,” “fitness score,” and “performance”. The first two scores are assigned by the authors of this review for every pipeline listed, while the performance score is a ranked-based approach to quantitatively compare all implemented pipelines used by any one reviewed paper (as most evaluated papers either try to compare their newly introduced pipeline to previously existing implementations or present different variants of their pipeline).

#### Online-score

While some pipelines have small latency requirements in order to be suitable for online studies and systems, others may require data batches of several seconds which makes them unusable in online settings. In order to quantify the suitability of a pipeline for an online BCI application, we assigned the scores 0, 1, or 2. The numbers are defined as follows:

**0:** The pipeline has been shown to be computationally inefficient with two or more seconds of delay.
**0:** The setting is not transferable to an online BCI scenario.
**0:** The pipeline assumes preconditions that cannot be met by online scenarios.
**1:** Is assigned if conditions 0 and 2 do not apply.
**2:** An online communication has been validated, e.g., sending a command to a device.
**2:** The pipeline has been validated with a latency below 1 s in an online setting.

The above definition was chosen because it was assumed that at least one needed information from above can be extracted from any given paper, no matter the paper structure, goals and focus. The idea was to assign as little pipelines as possible to category 1.

#### Fitness-score

Another important criterion, the “fitness score”, is needed to quantify the pipelines' fitness for being used in a BCI system. While motion artifact correction is crucial for a BCI, some pipelines only remove contaminated channels or time windows, resulting in data loss or unrealistic assumptions that may not be applicable in real-world scenarios. To quantitatively assess the suitability of a pipeline for a BCI application in a realistic context, we assigned scores of 0, 1, or 2 to evaluate the fitness of the pipeline.

The scores are defined as follows:

**0:** The pipeline has not been demonstrated to work with any real data.
**0:** The pipeline assumes preconditions that cannot be met by real-world scenarios, e.g., the pipeline requires an additional fNIRS measurement which is not ideal for building a BCI system.
**0:** Channels, trials, epochs, or windows that were contaminated with motion artifacts were removed.
**0:** The setting is not generalizable to the intended target population.
**0:** The pipeline does not filter motion artifacts.
**1:** Is assigned if conditions 0 and 2 do not apply.
**2:** The pipeline has been validated in real-world applications.
**2:** The pipeline corrects motion artifacts as they occur quantitatively and qualitatively.

The above definition was chosen because it was assumed that at least one needed information from above can be extracted from any given paper, no matter the paper structure, goals and focus. The idea was to assign as little pipelines as possible to category 1.

#### Mean rank-score

The third score quantifies how well a pipeline is suited to correct motion artifacts from EEG data based on the paper's direct comparisons (comparison results of the original authors). As the investigated papers are using different evaluation metrics, data recordings, and setups, it is not possible to compare the results from one paper with those from another directly. Results from different pipelines can only be compared if the same metrics, data recordings, and experiment setup are used. Moreover, many evaluations are based on distributions across trials, iterations, or study participants, which yields distributions for each pipeline's rank. In these cases, the significance of the distributions should be compared.

To address the heterogeneity among the presented pipeline comparisons, a rank-based approach was utilized. Pipelines with equal evaluation metrics, data, setup, and paper origin were ranked and normalized between 0 and 1. When comparing distributions, ranks were assigned based on the significance of differences, with the closest significance level to α = 0.05 chosen in case of multiple levels. Insignificantly different pipelines were regarded as ties and evenly broken. Subsequently, the ranks were arithmetically averaged for each unique pipeline within each reviewed paper. The resulting mean rank score falls within the interval of [0.0, 1.0]. Only methods from the categories “Filter,” “Aggregation,” “Decomposition,” “Artifact Detection,” “Artifact Correction,” and “Special Methods” were considered to identify unique pipelines. Fine-grained details, such as exact filter cut-off frequency, window width, or optimization criteria for ICA, were omitted, as they were not consistently mentioned on the same level of granularity across all investigated papers.

###  Quality appraisal and risk of bias

**Figure 2:**
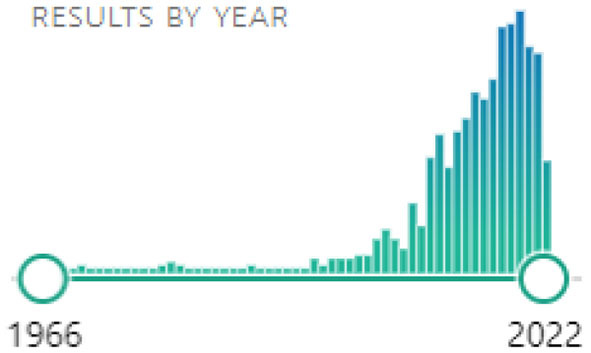
Pubmed search histogram from the year 1966 to 2022.

Considering the heterogeneity of the evaluated articles, we defined the relevant sections for applying the filter rules as: “Abstract,” “Methods,” and “Results”. Scores were given from 0 (no fit) to 2 (strong fit). Note that we do not rank the quality of the publication here, but the fit of the work to the defined goal of finding methods for motion artifact reduction suitable for online BCI experiments. Our search terms also provided us with many mismatch results dealing either with completely unrelated EEG topics or with artifact removal strategies that did not focus on motion artifacts, but other types of artifacts instead (e.g., non-physiological sources, eye saccades, electrocardiogram, stimulation artifacts, …). It is important to note that through the open access filter rule we discarded many of the older papers, in favor of newer literature, as can be seen from the final search histogram (see Figure 2):

This review, therefore, contains a bias toward newer publications, which was intended by the authors.

## Results

This section provides an overview of the authors, institutes, countries, journals, programming languages, used software, open code policies, and evaluation metrics that had the highest impact on the research area covered by this paper. Additionally it contains information on study design, participants, BCI paradigms, used methods and algorithms, descriptive analysis, method impact, electrode setups, EEG systems and ground truth sensors. The section is grouped into the following subsections:

- Demographics metadata (authors, institutes, countries, journals)
- Technology metadata (programming languages, software, open code policies and evaluation metrics)
- Study selection and taxonomy (subjects/participants, BCI paradigms)
- Methods for motion artifact removal (methods, descriptive analysis, method impact)
- Hardware for motion artifact removal (electrode setups, EEG systems, ground truth sensors).

###  Demographics metadata

The author with the highest number of publications was D.P. Ferris who was an author or co-author of eleven papers. Following him, W.D. Hairston contributed to seven publications, while P. König, S. Makeig, and F. Raimondo each had four papers. Furthermore, 21 authors contributed to three papers, 53 authors to two papers, and 266 authors to a single contribution.

**Figure 3:**
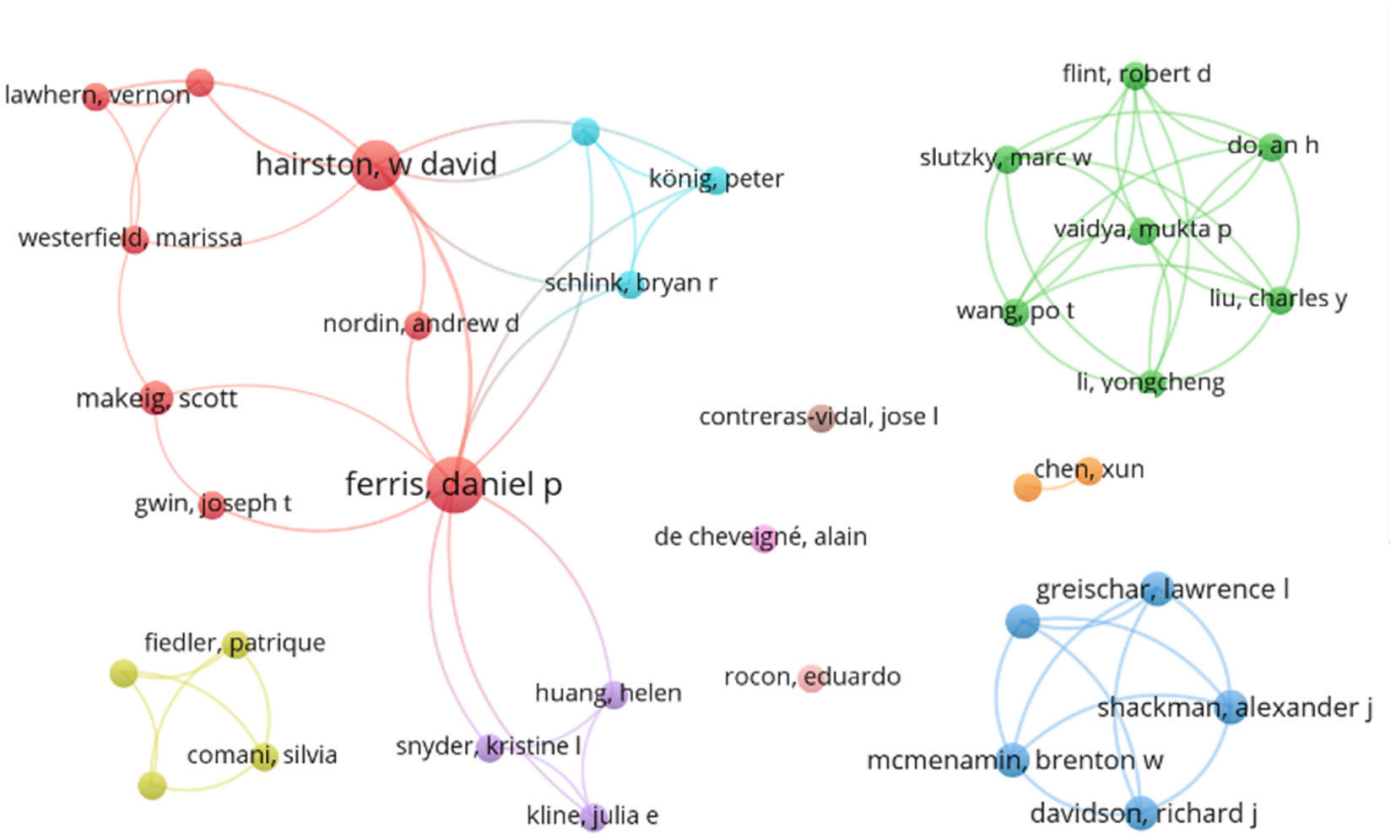
Reference map of all authors that contributed to at least two publications. Connections between authors correspond to co-authorships.

Figure 3 illustrates an authorship map of all authors that contributed to at least two reviewed publications, as first or co-author. The size of the points refers to the number of publications of the specific author, the connection represents a co-authorship. In particular, D.P. Ferris and W.D. Hairston have a research network involving multiple institutes and co-authorships.

In terms of institutions, the US Army Research Laboratory was associated with eight publications, followed by the University of Michigan with six, the University of California San Diego with four, and the University of Florida with three. Several other institutions contributed to two or one publication. For authors with multiple affiliations, each institution was counted separately.

The countries with the highest number of first authorship were the USA (31), Germany (13), China (8), Spain (7), France (6), Canada (5), UK (4), Italy (3), and India (3). In total, 33 countries contributed to the publications examined in this systematic literature review.

**Figure 4:**
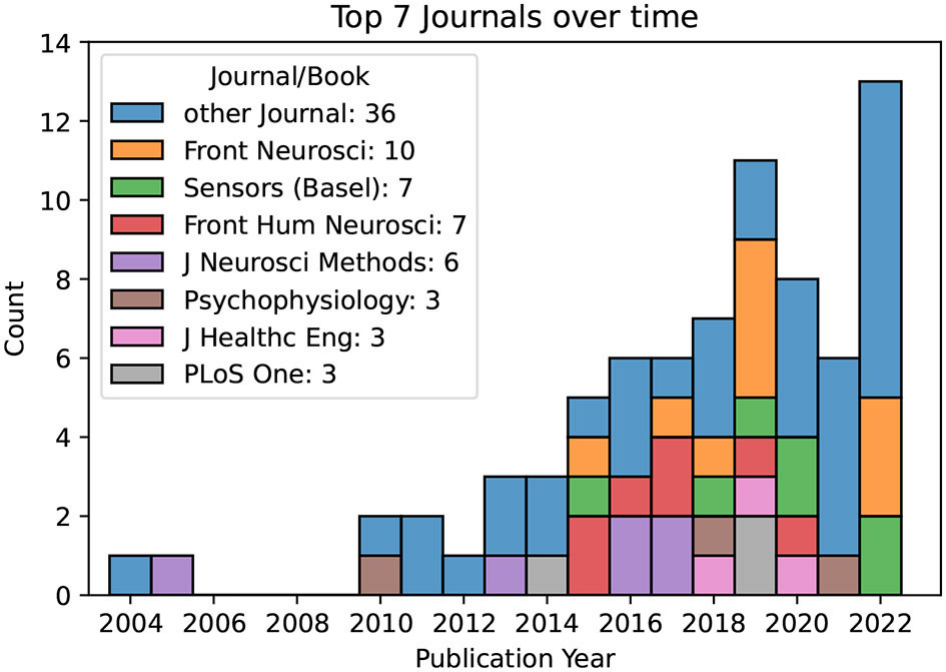
The most common Journals over time. Only four Journals contribute more than three publications to the subject of interest.

Lastly, we note that the top journals in terms of the number of publications included in our review were Frontiers of Neuroscience (10 publications), Sensors (Basel) (7), Frontiers in Human Neuroscience (7), Journal of Neuroscience Methods (6), Psychophysiology (3), Journal of Healthcare Engineering (3), and PLoS One (3). All other Journals contribute a total of 36 papers. Figure 4 illustrates the trend of the most common journals in this systematic literature review over time.

###  Technology metadata

Several open datasets were used by the authors of the evaluated articles, including:

- Temple University EEG Corpus (https://isip.piconepress.com/projects/tuh_eeg/html/downloads.shtml)
- SEED (https://bcmi.sjtu.edu.cn/home/seed/index.html)
- PhysioNet (https://physionet.org/content/?topic=eeg).

Open datasets provide a valuable resource for researchers to develop and evaluate new algorithms for EEG analysis. Two crowdsource label platforms were used by the authors:

- ALICE (http://alice.adase.org/)
- ICLabel (https://labeling.ucsd.edu/tutorial).

They aim to improve the labeling of EEG artifacts through the collective knowledge of several experts. Currently, both platforms focus on labeling independent components, which are created from the family of ICA methods (also see Hyvärinen and Oja, 2000; Delorme et al., 2007). These increasing datasets and labeling platforms can be used to train machine learning models that can further automate the classification of EEG artifacts.

**Figure 5:**
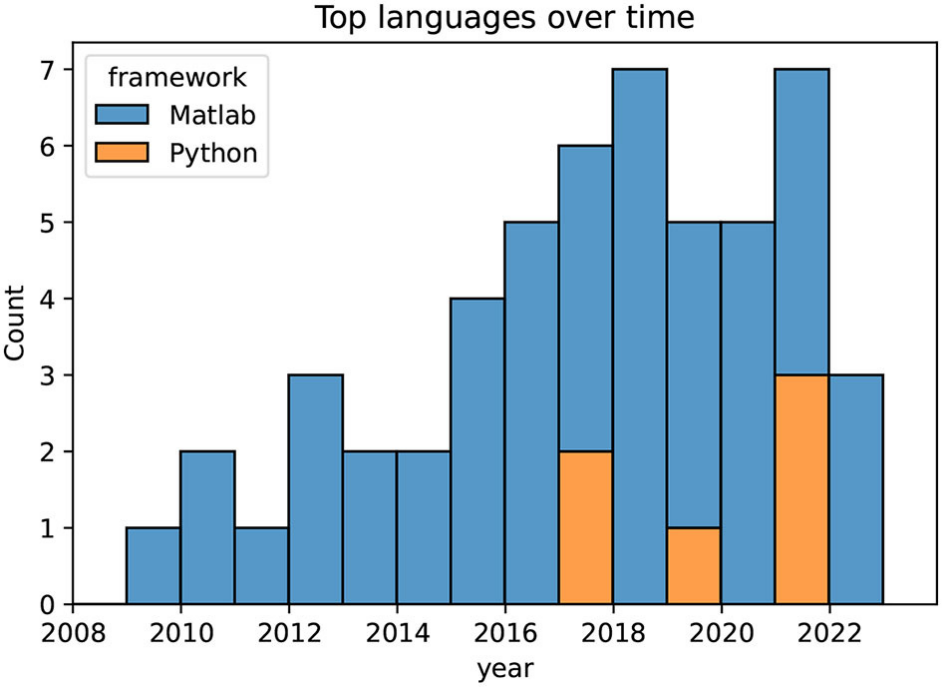
Programming languages over time, depicting a clear dominance of Matlab. Publications with no or unknown languages are omitted.

In Figure 5, the two most common programming languages for the pipelines are illustrated per year. The boxplot starts from 2009, as only a limited number of three papers were included in the review from before that year. The Figure depicts a clear dominance of Matlab (The MathWorks, USA), while three of seven implementations are in Python (Python Software Foundation, https://www.python.org/) in the year 2021. As the EEGLab software (Delorme and Makeig, 2004) is based on the Matlab language, pipelines implemented using EEGLab are also listed as Matlab. All pipelines for artifact mitigation and correction in the reviewed publications were implemented in either Matlab or Python.

For statistical analysis, some studies also used SPSS (IBM SPSS Statistics, 2023), Statistica (StatSoft Inc., 2023), or R (R Core Team, 2023). Various software tools were used for modeling the brain, including Neuroscan (Compumedics), Eevoke (ANT Neuro), BEAPP (Batch EEG Automated Processing Platform), BESA Dipole Simulator (MEGIS Software GmbH), Spike2 (Cambridge Electronic Design), SystemPlus (Micromed), BrainRecorder (Brain-Products GmbH), and E-Prime application suite (Psychology Software Tools, Inc).

In addition, VS.NET, Harmonie (Stellate), Persyst v12 (Persyst GmbH) and TracerDAQ software (National Instruments) were used for the experimental paradigm design and analysis. For motion capture systems or similar functionality, the software Visual-3D, FaceLAB (eye tracking system) and Vicon Nexus (Oxford, UK) were employed.

**Figure 6:**
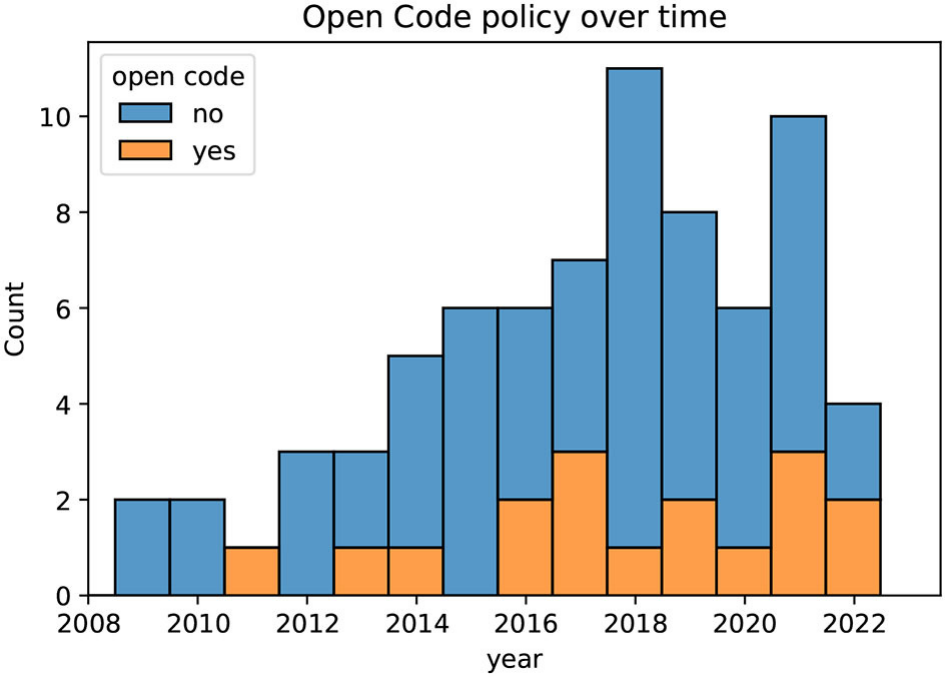
For each year since 2008, the open code policy of the papers is depicted.

Figure 6 illustrates the open code policy in the publications reviewed in this study. The figure shows the fraction of papers that make their code publicly available grouped for each year. Prior to 2016, only some authors shared the details of the pipeline implementations used in their experiments. However, in 2022, two out of four publications provide their code. An increase in open code policy is a positive trend for the scientific community, as it allows for greater reproducibility and transparency of research results.

**Figure 7:**
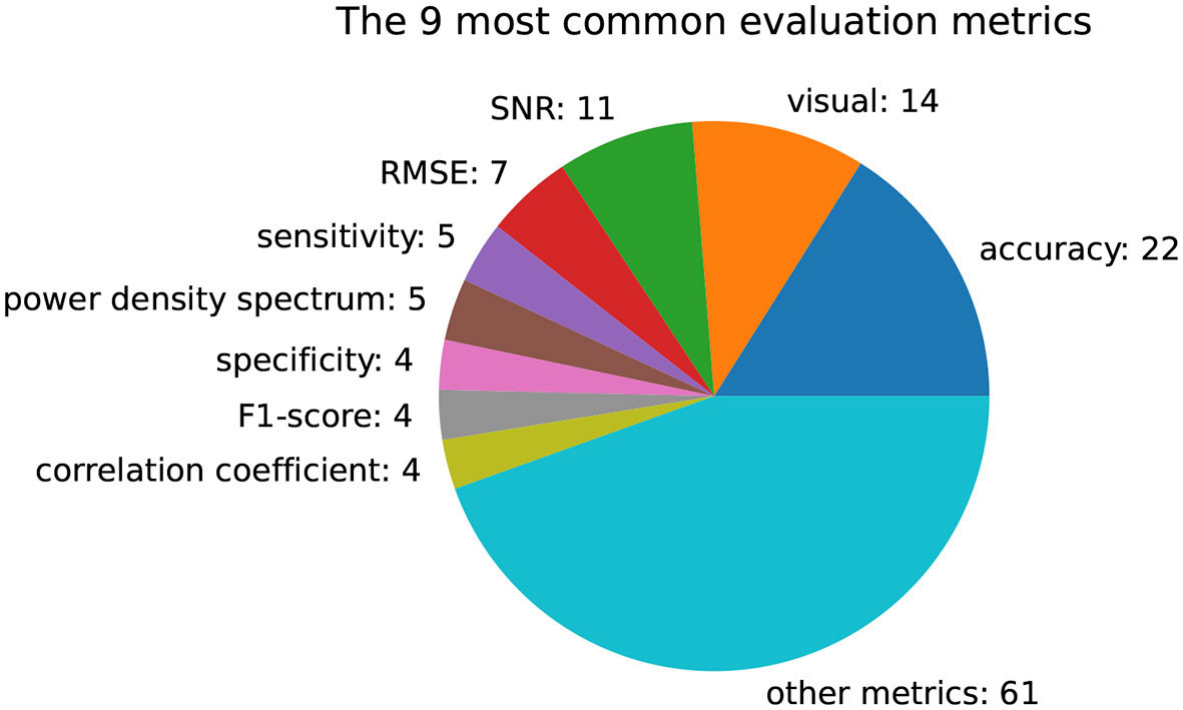
Number of publications using a specific evaluation metric. The majority of used metrics are used less than four times, while the accuracy, visual proof, and signal-to-noise ratio (SNR) are the most common ones.

Figure 7 presents a visualization of the most common evaluation metrics used in the studies included in this review. As there is currently no widely accepted standard metric for evaluating EEG artifacts, a high number of different metrics are applied. Out of the 77 publications analyzed, 22 of them compare their pipelines based on an accuracy metric. This accuracy metric is not limited to downstream classification tasks such as mental gesture classification, but also includes the classification of artifact presence or type. In the absence of ground truth brain signals, 14 contributions rely on a qualitative visual assessment or a quantitative signal-to-noise ratio (SNR) for evaluation purposes. The evaluation metrics that are used less than four times are aggregated into the “other metrics” category.

###  Study selection and taxonomy

**Figure 8:**
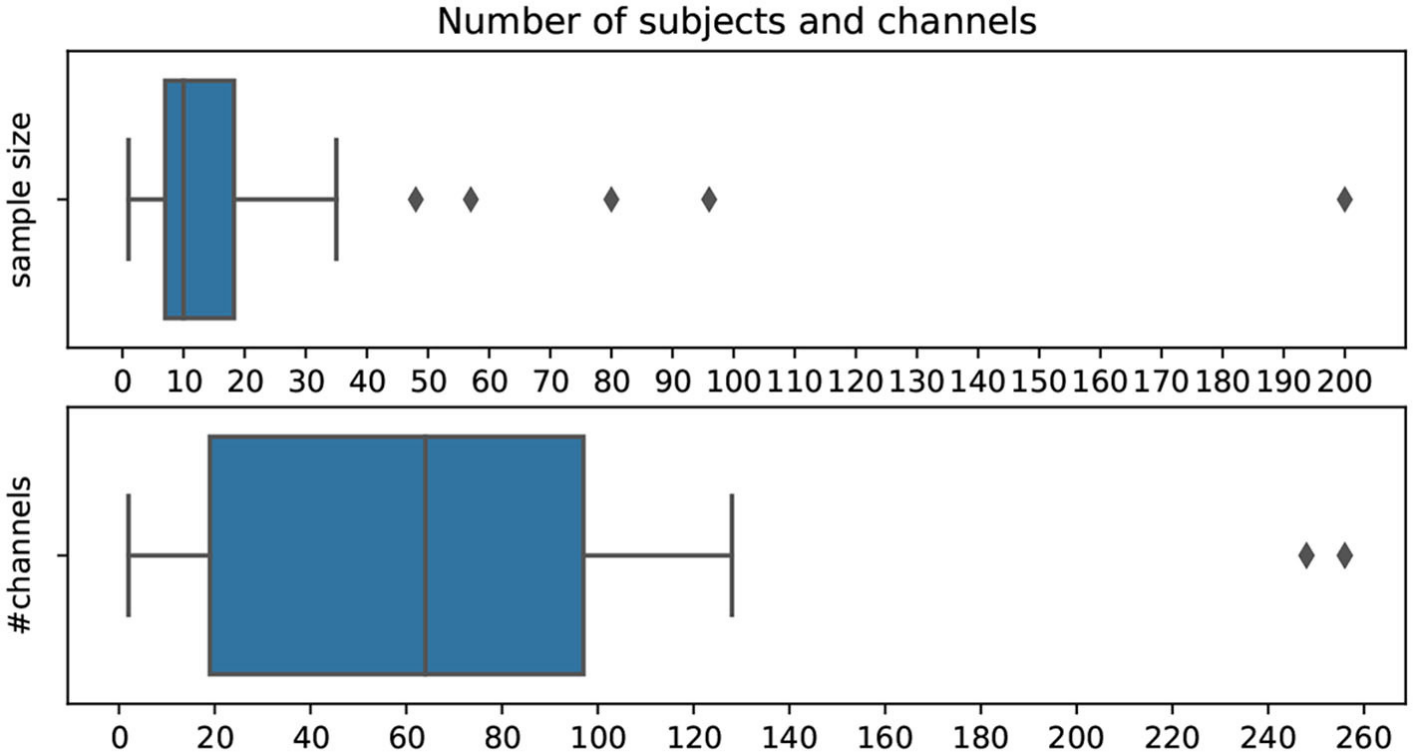
Number of subjects for each dataset and number of used EEG channels per publication.

In Figure 8, the distribution of the number of subjects and channels used in the pipelines per publication is shown. The mean number of subjects per analyzed dataset is 16.4, with the majority of studies not having more than ten subjects. Only 7.6% of datasets included 30 or more participants. Additionally, half of the studies use >64 EEG channels. As EEG attempts to become more easily mountable, many publications investigate settings with only a small number of channels.

**Figure 9:**
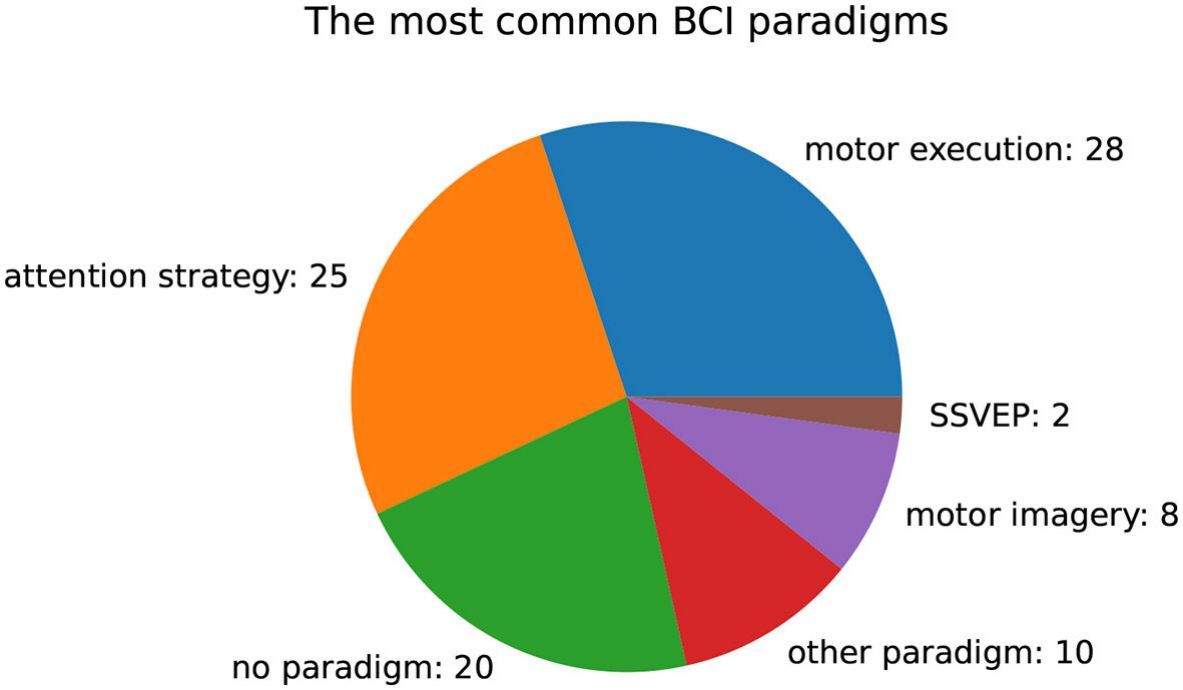
Number of used BCI paradigms in publications.

Figure 9 presents an overview of BCI paradigms used in the investigated cohort of papers. Among the publications analyzed, motor execution (28) and attention strategies (25) were the most commonly employed paradigms. Twenty publications focused on mitigating or correcting artifacts and did not use any specific paradigms. Additionally, we found that motor imagery (8) and steady-state visually evoked potentials (SSVEP) (2) were used in some cases. In 10 cases, a different paradigm was applied, that occurred only once.

###  Methods for motion artifact removal

#### Methods

Within the reviewed publications, a total number of 303 pipelines for artifact treatment, composed by various methods, were presented. These methods can be categorized into several categories, such as filters, aggregation methods, decomposition methods, artifact detection methods and specialized methods and subpipelines. In addition, classification methods for a downstream task on cleaned and corrected EEG data were found, that are not investigated in more detail within the scope of this paper.

Regarding the filters, we found that 261 out of the 303 pipelines use frequency filters such as high-pass, low-pass, band-pass, band-restriction, and notch-filters. Adaptive filtering (AF) was used in 24 pipelines, Moving average (MA) in 10 and 20 pipelines used other filters such as smoothing algorithms like e.g., the Savitzkey-Golay filter.

Epoching the measurement into time-constrained batches (127) and generating features within these windows (104) are grouped into the category Aggregation methods. Among all generated features the most common ones were: Kurtosis, standard deviation, several features of the power density spectrum, correlations between channels or with artifact templates, autocorrelation, entropy, fractal dimension, the spatial average difference (SAD), spatial eye distance (SED), and Myogenic identification feature (MIF).

The category “Decomposition” also includes several forms for spectral decomposition as well as spatial blind source separation algorithms. A Fourier transformation was applied in 31 publications and the Wavelet transform in 50. Independent component analysis (ICA) was used in 114 out of 303 pipelines, canonical correlation analysis (CCA) in 25, Principal Component Analysis (PCA) in 44, empirical mode decomposition (EMD) in 26, and common spatial patterns (CSP) in 9. We found other methods that are called Welch power spectral density, Lomb-Scargle periodogram, Nonnegative Matrix Factorization (NMF), t-distributed stochastic neighbor embedding (t-SNE), spatio-spectral decomposition (SSD), joint blind source separation (JBSS), independent vector analysis (IVA), singular spectrum analysis (SSA), SOBI, ERICA, AMUSE, Auto-regression, Local and Weighted Average Reference, Riemann Kernels, SNS and Phase Lag Indexing.

For the detection of artifacts, Linear Regression was used in seven pipelines, Discriminant Analysis (DA) in 15, support vector machines (SVM) in 12, Spatial spherical splines in 16, and Gaussian Mixture Models (GMM) in four. Additionally, 25 other methods were used for this purpose. In order to correct artifacts, in only nine publications an Autoencoder was used, and in four a GAN. Artifacts were frequently corrected by decomposing the signal, detecting artifactual components, and removing them during the signal reconstruction from the components. Some methods and subpipelines specialized for EEG were grouped into an additional category, in particular ADJUST (20), FASTER (16), MARA (9), HAPPE (6), and ERASE (4).

#### Descriptive analysis

**Figure 10:**
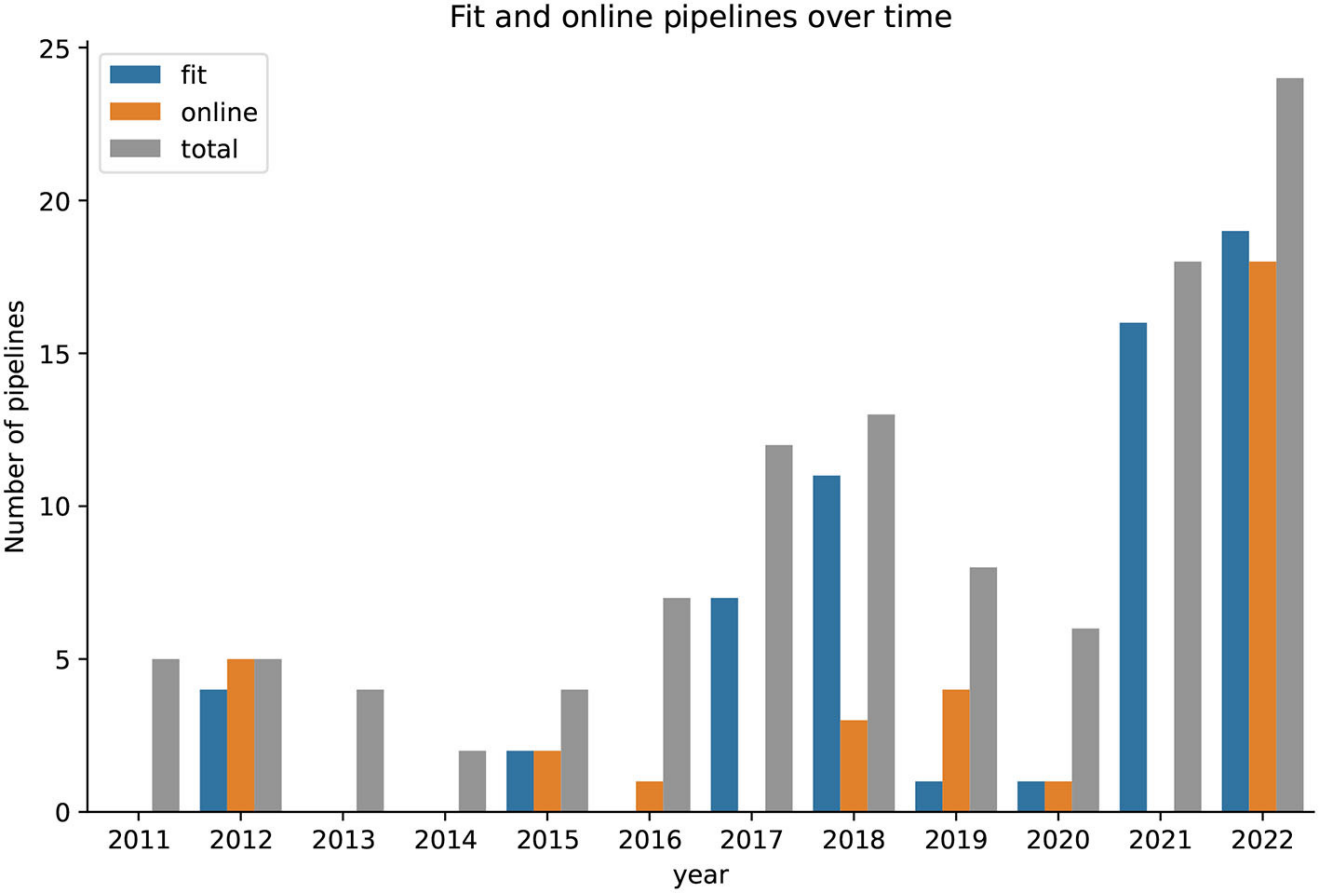
The number of totally proposed pipelines per year since 2011 with fractions of pipelines that are fit for a use case with BCI and are online capable. In 2021, none of the proposed pipelines were online capable.

Figure 10 presents the fitness of the proposed pipelines for BCI application and their online capabilities over time. The number of pipelines achieving a score of 2 in the respective scale, as defined in previous sections, was summed up for each year. As few papers were published before 2010, the timeline starts in 2011. The results suggest that the fitness of the proposed pipelines are unstable over the years.

**Figure 11:**
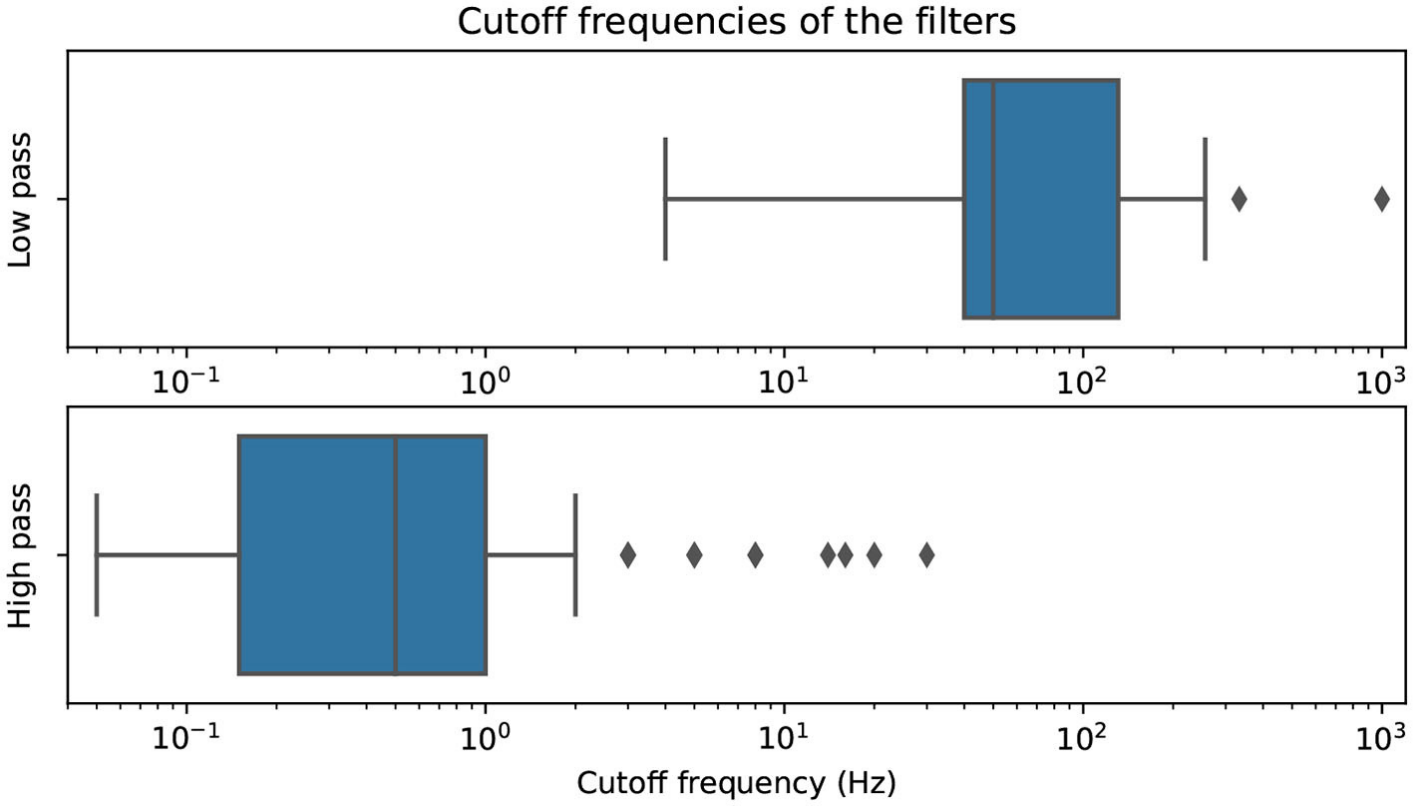
Cutoff-frequencies of all filters, split into low-passes and high-passes (both with band-pass bounds) and plotted on a logarithmic scale.

Figure 11 presents the distribution of commonly used cutoff frequencies for EEG filtering. The upper bound of band-pass filters is included in the low-pass filter distribution and vice versa. Our analysis shows that the interquartile range for high-pass filters is between 0.15 and 1 Hz, indicating the need for drift correction in the signals. Additionally, many authors filter out frequencies higher than the typical electrical powerline frequency using low-pass filters.

Notably, notch and band-restrict filters, commonly used to remove specific frequency bands, are not visualized in the boxplot. These filters serve a specific purpose and do not contribute to the overall distribution of cutoff frequencies.

**Figure 12:**
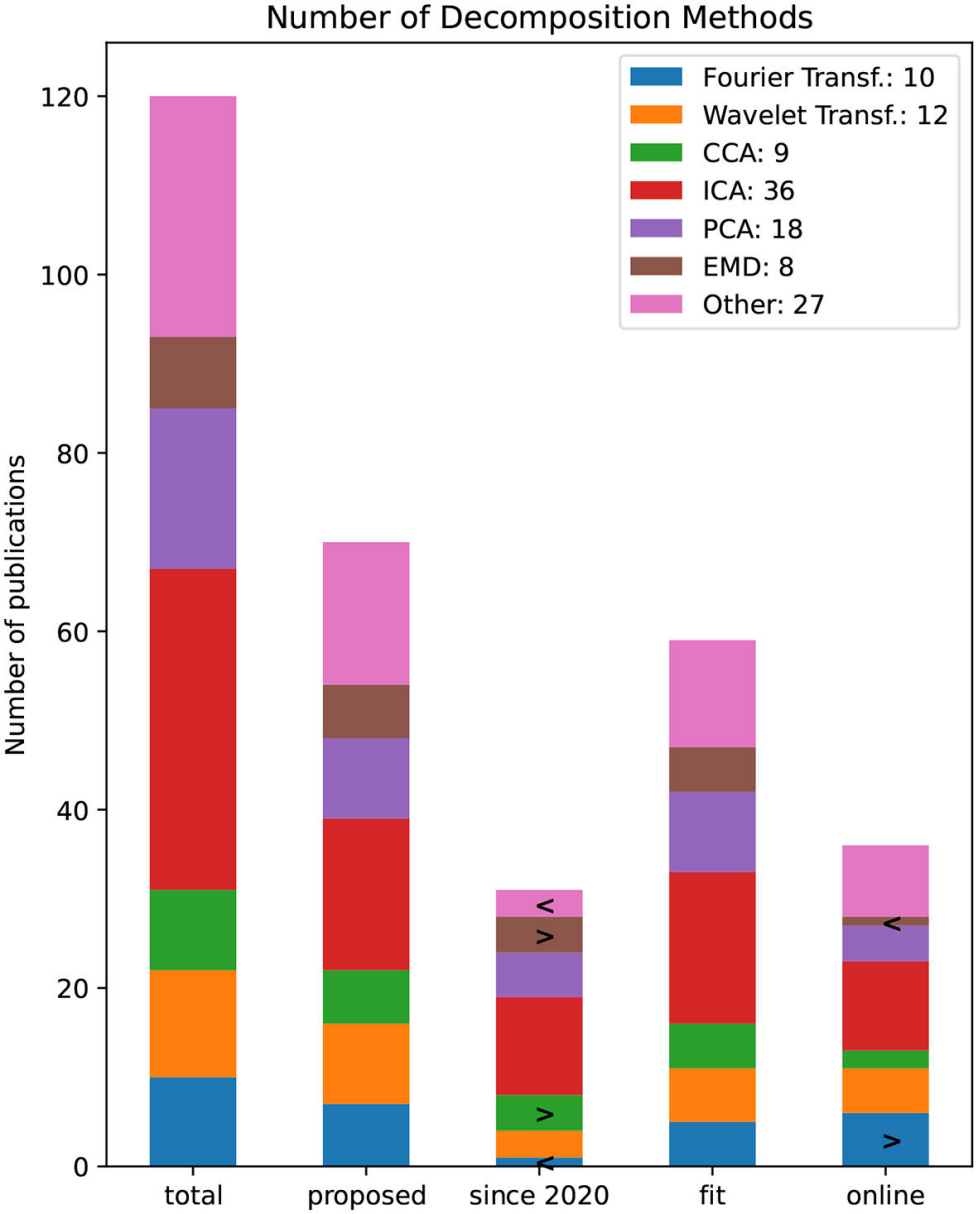
Number of decomposition methods by the number of publications. The bars refer to all publications (since 2020) and proposed pipelines that are noted to be fit for BCI rsp. online capable.

Figure 12 depicts the number of papers that use selected decomposition methods. All bars other than the “total” bar have applied a specific filter criteria and therefore are a subset of the “total” bar. The filtered bars show all newly proposed, all since 2020, and pipelines that are noted to be fit for BCI respectively online capable. The most common decomposition method is ICA used in 36 publications followed by PCA in 18. A total of 22 publications decomposed the EEG signal within the time domain using a Wavelet or Fourier transformation. If a method occurs significantly often, this method is marked with the symbol (>) indicating that the Null-hypothesis can be rejected to a significance level of α = 0.05 using the bootstrap resampling method (Efron, 1983). A second bootstrap estimation is performed to test if a method occurs significantly less in a filtered bar, thus noted with the symbol >.

Since 2020 canonical correlation analysis (CCA) and the empirical mode decomposition (EMD) method was significantly often used. While EMD seems not to be suitable in an online scenario, the Fourier Transformation or some implementation of it might be.

**Figure 13:**
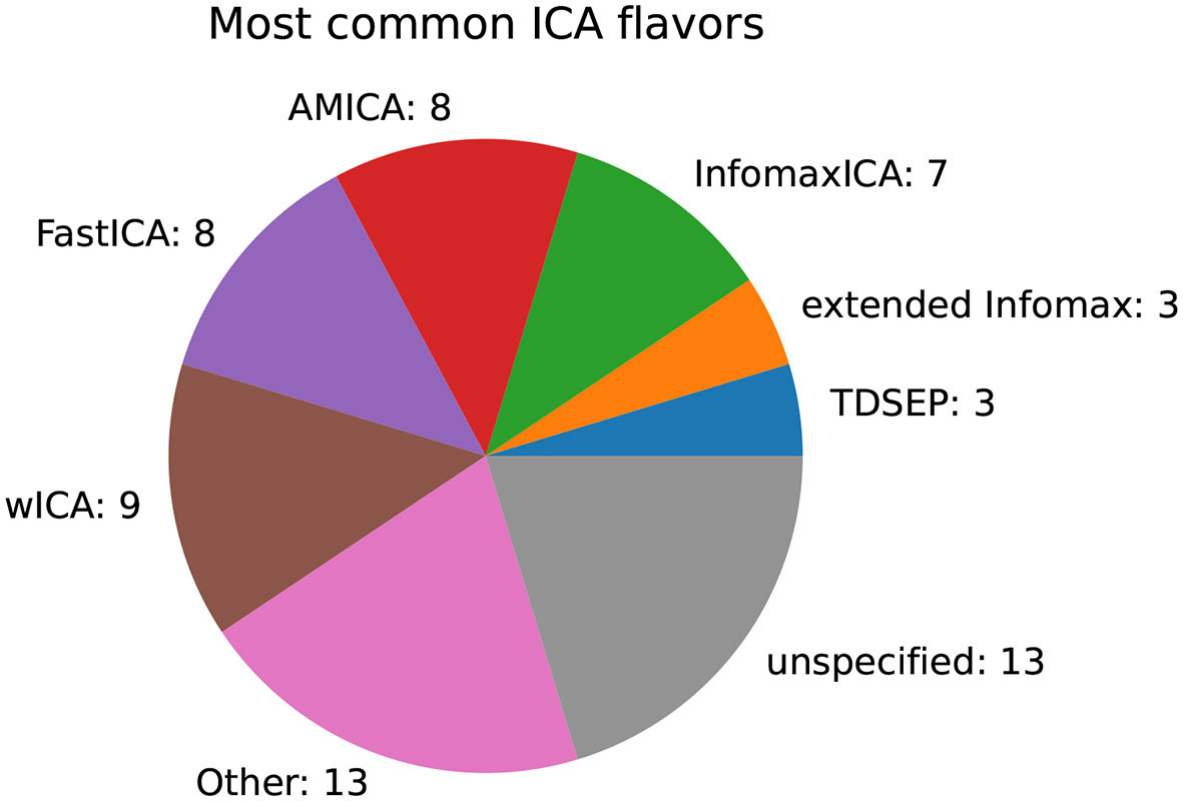
Number of ICA variants by the number of publications.

For the most common family of decomposition methods, namely ICA, an investigation of the variants used is of interest. Figure 13 summarizes the most common variants. Most authors applied, wICA (wavelet), FastICA, AMICA, and InfomaxICA. In 13 publications, the variant was not specified.

**Figure 14:**
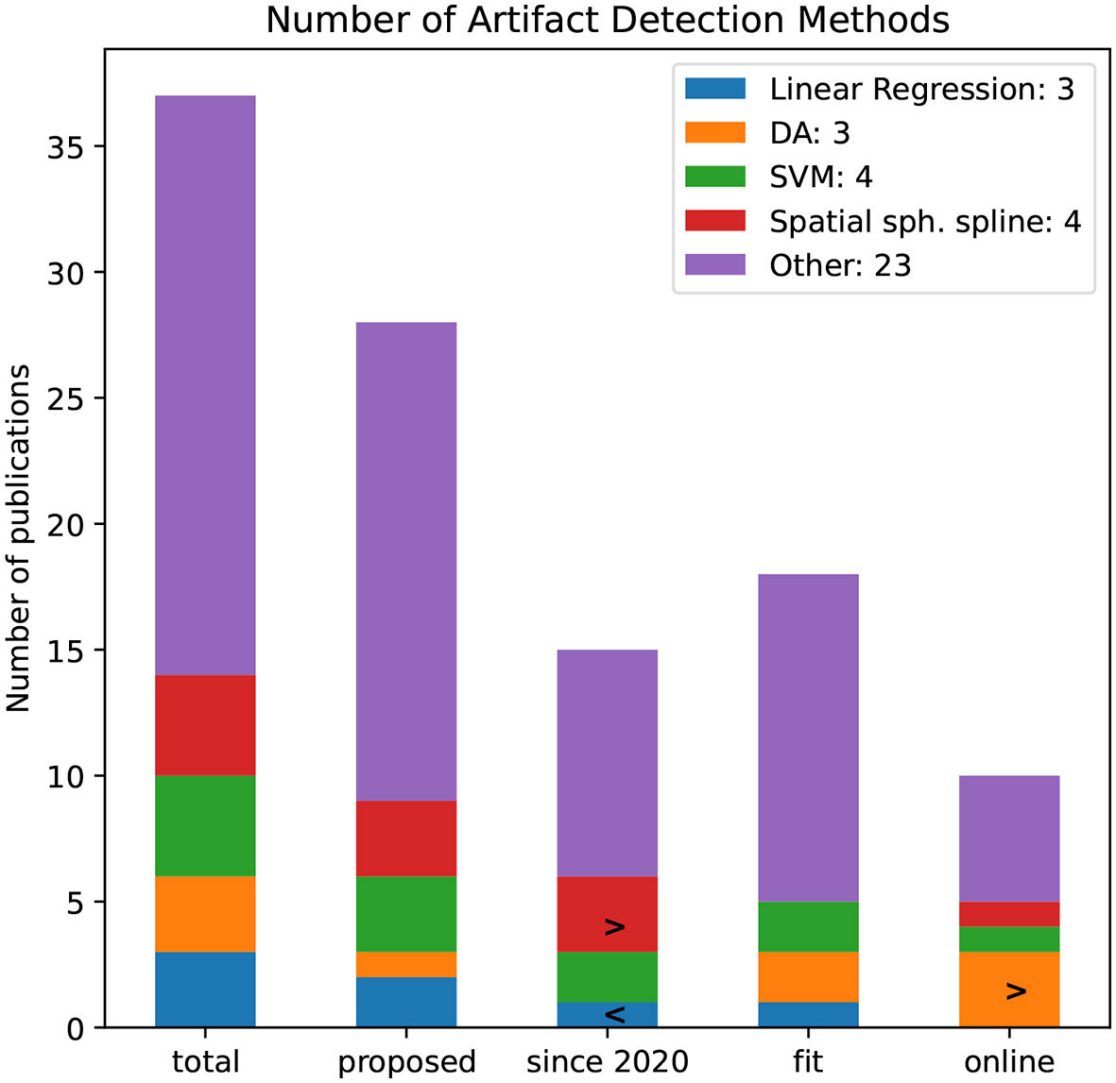
Number of artifact detection methods by the number of publications. The bars refer to all publications and proposed, since 2020, and pipelines that are noted to be fit for BCI rsp. online capable.

The artifact detection is applied to decomposed components or views of the original EEG signal. Figure 14 illustrates the number of papers that are using selected artifact detection methods. Methods that are not used in more than two publications are grouped into the “Other”-category. This category is with 23 publications by far the largest class, indicating that no standardized artifact detection method has been established.

Similarly, the total number of artifact detection methods is filtered whether they are newly proposed, used since 2020, fit for BCI application, and online capable. Discriminative Analysis (DA) was not applied in reviewed publications since 2020 but seems to be online capable. Since 2020, more authors applied spatial spherical splines.

**Figure 15:**
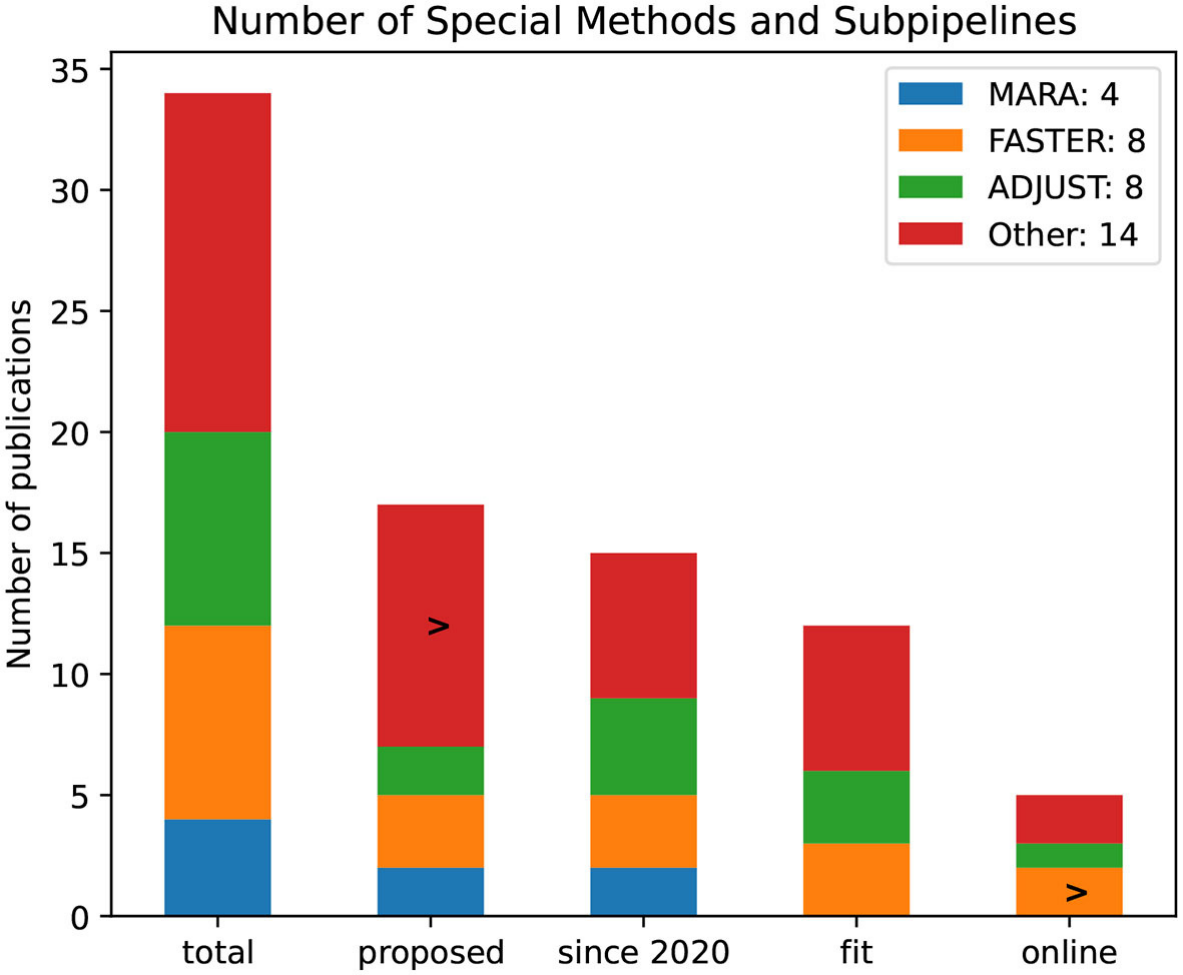
Number of specialized EEG methods and subpipelines by the number of publications. The bars refer to all publications and proposed, since 2020, and pipelines that are noted to be fit for BCI rsp. online capable.

Many authors rely on the usage of methods and subpipelines specialized for EEG signals, as illustrated in Figure 15. Eight publications use the ADJUST algorithm and the FASTER subpipeline. The method MARA was used in four publications, but none of the introduced pipelines seems fit for a BCI use case or online capable. The FASTER method, as the name suggests, seems to be fit for an online application.

#### Impact of the methods

Table 2 summarizes the effect of using selected methods within a pipeline. The table presents the number of authors and pipelines that used or not used each method respectively, and its impact on the mean normalized rank across all pipelines and publications. We also show the absolute difference in rank between pipelines that used or omitted a particular method. The right column of the table presents the statistical significance of the improvement in pipeline rank achieved by using a particular method expressed in terms of the *p*-value. To calculate the *p*-value, we performed an exact permutation test with 10,000 runs (by using the method described in Ernst (2004).

**Table 2:** For each method in a pipeline, the number of papers and pipelines using or not using it are depicted with their respective mean normalized rank.

	**With/without Method**			**diff**.	**p-value**
		**pipelines**	**mean norm. rank**		
Linear Regr.	3 / 51	45 / 737	0.252/0.613	−0.3606	**0.0018**
AF	3 / 51	65 / 717	0.331/0.604	−0.2733	**0.0002**
ADJUST	7 / 51	50 / 732	0.407/0.599	−0.1915	**0.0012**
CCA	9 / 49	55 / 727	0.459/0.625	−0.1656	**0.0042**
ICA	28 / 41	283 / 499	0.511/0.673	−0.1624	**0.0122**
SVM	3 / 51	63 / 719	0.471/0.605	−0.1338	**0.0458**
Epoching	26 / 33	454 / 328	0.524/0.658	−0.1333	**0.0154**
EMD	7 / 49	70 / 712	0.497/0.619	−0.122	**0.0414**
CSP	3 / 50	20 / 762	0.492/0.600	−0.1081	0.1316
Feature Ext.	17 / 39	393 / 389	0.513/0.616	−0.1024	0.0960
Sph. spline	3 / 50	56 / 726	0.524/0.602	−0.0779	0.4638
FASTER	6 / 50	45 / 737	0.527/0.597	−0.0707	0.2658
MARA	4 / 51	12 / 770	0.552/0.597	−0.0446	0.7191
Wavelet Tr.	8 / 48	114 / 668	0.577/0.608	−0.0309	0.6863
PCA	12 / 50	91 / 691	0.660/0.612	0.0484	0.4500
PCC	2 / 51	22 / 760	0.687/0.598	0.0892	0.4761
Fourier Tr.	8 / 44	111 / 671	0.697/0.584	0.1128	0.0924
MA	2 / 50	51 / 731	0.753/0.598	0.155	0.2066
filter	46 / 10	582 / 200	0.629/0.454	0.1749	**0.0068**
DA	3 / 49	52 / 730	0.836/0.588	0.248	**0.0314**

Additionally, the *p*-values show the significance of the impact of using a method in a pipeline. *p*-values > 0.05 are marked in bold because it means rejection of null hypotheses (< 0.05 for significance level is an accepted convention).

Our results suggest that several methods, including Linear Regression, Adaptive Filtering (AF), ADJUST, CCA, and ICA, contribute significantly to improving the mean normalized rank. In contrast, methods like PCA, Pearson Correlation Coefficient (PCC), and Discriminative Analysis (DA) even had a negative effect on the pipeline's performance. One interesting finding is that Wavelet performed better than Fourier transformation. This effect may be due to Wavelets' higher temporal resolution, which may be more important than the precise frequencies of EEG signals.

###  Hardware for motion artifact removal

Most authors use the traditional Ag-AgCl electrode setup with placements according to the typical 10–20 system setup. Electrodes are either dry, water-tab-based, or gel-based. Typical reoccurring vendors for electrodes were g.tec, ANT-Neuro, BrainProducts and Biosemi. More custom untypical electrode setups featured for example:

- MEMS (dry micro-electromechanical sensors)
- 3D printed PWS electrodes coated with poly polystyrene sulfonate (PEDOT:PSS)
- 3D Printed dry concentric Electrodes
- Multipin Polyurethane electrodes.

Caps and amplifiers were more heterogenous than used sensors, but g.tec, ANT-Neuro, BrainProducts and Biosemi reoccurred often here as well. Additionally, authors used systems from companies like BrainWave (Medi Factory, Netherlands), WaveGuard (ANT-Neuro, Netherlands), Cognionics (USA), Mindo (National Chiao Tung University), Electro-Cap International Inc (USA), EasyCap (Germany), Neuracle (China), Neuroelectrics (Spain), Brain-Net EMSA (Brazil), Plexon Inc. (USA), Electrical Geodesics Inc. (USA), LaMont Medical (USA), Neuromag (Mexico), Natus Medical (Canada), Nihon-Kohden (Japan), Spes Medica (Italy).

Especially interesting were two novel device setups from Snyder et al. (2015) and Nordin et al. (2020). The idea for both publications was to decouple true brain activity from measured external motion artifacts by artificially blocking and/or creating the true brain component part. In Snyder et al. (2015) a novel 3 layer system was proposed: (1) Silicon swim cap, (2) simulated scalp, and (3) EEG system. In Nordin et al. (2020) a novel 2-layer EEG system was proposed: (1) 128-scalp EEG electrodes and (2) a custom conductive fabric cap which approximately matched the resistivity of human skin. The dual-layer EEG from Nordin20 simultaneously recorded human electrocortical signals and isolated motion artifacts using pairs of mechanically coupled and electrically independent electrodes and a custom conductive fabric cap.

Additionally, many diverse hardware setups for ground-truth measurement of the EEG were used, e.g.,:

- Bi-lateral force plates in a treadmill
- SMU (source measure unit)
- IMU (inertial measurement unit)
- EMG (electromyogram)
- EOG (electrooculogram)
- ECG (electrocardiogram)
- Accelerometers
- Gyroscopes
- Camera systems.

## Discussion

###  Study metadata

The results revealed the most prolific authors, institutions, and countries contributing to the field, with D.P. Ferris and W.D. Hairston being the authors with the highest number of publications. The US Army Research Laboratory, University of Michigan, and University of California San Diego were among the top institutions. The USA, Germany, and China were the countries with the highest number of first authorships. The study also identified the top journals publishing articles in this field, such as Frontiers of Neuroscience, Frontiers in Human Neuroscience, and Sensors (Basel). Open datasets and crowdsource label platforms were introduced as valuable resources for researchers, and Matlab was found to be the dominant programming language used in the implementation of artifact mitigation and correction pipelines.

###  Methods for motion artifact removal

A total of 303 pipelines from the reviewed publications were analyzed, which included filters, aggregation methods, decomposition methods, artifact detection methods, and specialized methods and subpipelines. Frequency filters, such as high-pass, low-pass, band-pass, and notch filters, were commonly used for artifact rejection and correction. The distribution of cutoff frequencies for EEG filtering showed that high-pass filters typically had cutoff frequencies between 0.15 and 1 Hz, indicating the need for drift correction, while low-pass filters commonly filtered out frequencies below the electrical powerline frequency.

Aggregation methods involved epoching the measurement into time-constrained batches and generating features within these windows, with common features including kurtosis, standard deviation, power density spectrum, and spatial correlations. Decomposition methods included Fourier transformation, wavelet transform, independent component analysis (ICA), and principal component analysis (PCA), among others. Methods such as Linear Regression, Adaptive Filtering, ADJUST, CCA, ICA, SVM, Epoching, and EMD improved the pipelines significantly while Moving Average, filters, and Discriminant Analysis decreased it.

###  Research gaps

Within this subsection, identified consensus and commonly known research gaps are presented and discussed.

#### General research gaps

All authors of the reviewed papers emphasize, that motion artifacts prohibit the usage of mental signals via EEG for BCI systems in the real world. Muscle artifacts originating from whole-body movements are more complex to handle than other EEG artifact types because it impacts the EEG in a broad frequency spectrum and a high amplitude. This leads to artifacts with amplitudes that are typically higher than those of the signal and which are present in a broad spectrum in the frequency domain. Therefore, the reduction of motion artifacts in an EEG is a nontrivial task (Gwin et al., 2010; Snyder et al., 2015; Symeonidou et al., 2018).

There is a lack of standardized preprocessing steps that are validated and include basic filtering of noise signals. Few theoretical or practical approaches were conducted that examined the effect of filtering methods on the latency and signal form of the mental EEG signals (Anders et al., 2020; Karpiel et al., 2021).

Within the reviewed papers, we found that many characteristics of phase-locked EEG signals are often only visible by averaging across multiple gaits, trials, and even subjects. The authors argue that these mean characteristics are “typical brain patterns” suited to be used as commands for BCI systems (Delorme, 2022). However, their high inter-subject and inter-trial variability shows that approaches based on averaged characteristic patterns might not help to build robust BCI systems (Kline et al., 2015; Nathan and Contreras-Vidal, 2016).

Additionally, many pipelines rely on methods with strong assumptions that might not be met in real-world conditions, e.g., a scarcity or homogeneity of artifacts cannot be assumed for diverse whole-body movements. For instance, Mur et al. (2019) mentioned that their pipeline requires a time epoch free of artifacts before and after each artifact as well as no bad electrode. Similarly, de Cheveigné (2016) mentioned that their pipeline does not work if an artifact affects multiple electrodes. In particular, the most present decomposition method in the review, ICA, has also strong assumptions that we will further discuss in the subsection “*The well known limitations of ICA*”.

Only a minority of the reviewed literature conducted benchmark testing of new or existing algorithms and pipelines. The most comprehensive benchmark testing was performed by Jas et al. (2017) who compared several pipelines on four different databases, multiple mental strategies, and system setups, for a total of more than 200 participants.

According to Grosselin et al. (2019) subject-driven classification performance needs long-lasting individual studies with longitudinal recordings to reach its full performance potential. The presented algorithm in Grosselin et al. (2019) is not subject-driven but the authors noted there could be a great optimization potential for the classification methods (LDA, SVM, kNN) by fine-tuning them on individual longitudinal recordings lasting weeks or months.

#### Ground truth problem

The field of EEG data analysis faces a significant challenge in separating non-neuronal motion artifacts from neuronal activity, as noted in several studies (Gwin et al., 2010; Snyder et al., 2015; Symeonidou et al., 2018; Delorme, 2022). The primary reason for this difficulty is the lack of a ground truth measurement of the pure brain signals, which makes it challenging to create and evaluate artifact removal methods. Some methods aim to correct simulated artifacts that were added to clean EEG segments to compensate this problem, but simulated artifacts do not represent the full range of real artifacts that can affect EEG recordings (Yong et al., 2012; Tamburro et al., 2018).

As a result, there is a lack of consensus on benchmarks for comparing the performance and usability of EEG systems, and no reliable quantitative metric for evaluating artifact removal methods has emerged (Delorme, 2022). In practice, many authors rely on the visual comparison, signal-to-noise ratio (SNR), or correlation to validate the quality of the artifact reduction method, but these practices have questionable scrutiny (Oliveira et al., 2016; Mur et al., 2019; Delorme, 2022). A comprehensive summary of the most common metrics applied in the reviewed papers is provided in Figure 7.

One approach to address the challenge of unavailable ground truth brain signals is to isolate electrodes from the head using a swimming cap (Kline et al., 2015; Snyder et al., 2015). However, the artifacts measured in the electrodes (with increased impedance) solely represent induced voltages, e.g., from cable movements, and do not include artifacts stemming from muscle activity. Some studies have shown that placing additional EMG electrodes over the face and neck muscles can be beneficial (Jas et al., 2017; San-Martin et al., 2018; Liu et al., 2019; Mucarquer et al., 2020; Nordin et al., 2020). For example, Mucarquer et al. (2020) validated the improvement of EEMD-CCA using EMG channels. Adding measured EMG artifact channels to EEG channels also improves ICA performance, as it learns and detects EMG contamination within EEG and forces EMG artifacts into a minimal number of independent components (Li et al., 2021). Different methods use different ways of mixing these EMG signals into EEG channels, and comparing EMG-added removal methods can help determine the optimal mixture of signals from EMG and EEG.

Single motor unit studies (focusing on one individual muscle or muscle group) with an attempted ground truth measurement have a very high value as they add knowledge of individual muscle contribution to the EEG system. There is a need for more studies targeting different muscles or muscle groups. In Yilmaz et al. (2014) we can find information on how the temporalis muscle impacts the EEG. Understanding EEG contamination on the level of individual muscle contribution helps identify new ways to detect and mitigate their effects. We have found very few papers in our research that try to separate EEG muscle artifact contamination into the contribution of individual muscles or muscle groups. In almost all other works, the muscle contamination (with the exception of eye movement) is seen as a summation of artifacts that get detected and removed as a whole. This approach, however, might be the wrong way to do it though, as Yilmaz et al. (2014) argues that the effect of single muscles on the EEG signal should be further examined, rather than all muscle contamination getting detected and removed as a whole. A full-body movement can be modeled as a composition of multiple individual muscles as part of a larger muscle group, but the specific artifacts present in each electrode show the summed up noise of all individual contributions. We have found no other papers that try to separate EEG muscle artifact contamination into individual muscles or muscle groups.

Another option for addressing this challenge is to optimize the parameters of a filter method based on a criterion for a downstream task, i.e., a task that does not aim to detect artifacts but to solve the actual problems for an application such as in a BCI system. In our literature review, 13 publications validated at least two pipelines with a classification downstream task (see the spreadsheet in the provided repository for more information). When following such an approach, it has to be taken into account, that the resulting parameters of the pipeline's methods are optimized only for a specific downstream task and not generically for all brain signals.

It should be mentioned, that in Winkler et al. (2011) the successful removal of artifacts and correctly detected outliers did not lead to better classification accuracy, potentially because the filtering removed characteristic brain signals essential for the downstream task. This suggests further investigating whether correctly detected artifacts can even always lead to better classification accuracy. Recently, Delorme (2022) showed that almost none evaluated pipelines for artifact reduction increased the performance significantly over multiple datasets.

In summary, only little empirical evidence is given, as to whether the pipelines for artifact reduction and correction only reduce noise and retain brain signal components required for a BCI system's downstream task. Methods for the reduction of motion artifacts could therefore be too aggressive and also filter mental signals among myogenic artifacts to a severe and unknown degree.

#### Method comparison

The reviewed literature lacks rigorous comparisons between existing methods for reducing motion artifacts in EEG data. Many papers compare their proposed method with a default pipeline without any artifact reduction or do not validate it against well-established and successful artifact reduction techniques. This raises concerns about the validity of the proposed methods as well as the state-of-the-art methods.

In particular, ICA or one of its variants is directly compared to CCA (or its variants) only in Chen et al. (2014) and Dai et al. (2021), and to PCA (or its variants, mostly ASR) only in Gordon et al. (2015); Arad et al. (2018), and Rosanne et al. (2021). In 77 investigated publications, there were neither direct comparisons between the de facto standard method ICA and a variant of EMD nor CSP. In addition to that, hardly any researcher compared their proposed pipeline with a more recent pipeline of other researchers that is successful for a similar use case. Nevertheless, multiple authors of the reviewed literature mention the importance of a quantitative comparison of proposed methods with existing successful methods in order to increase the comparability of the pipelines (Yong et al., 2012; Frølich and Dowding, 2018; Karpiel et al., 2021; Saba-Sadiya et al., 2021; Fló et al., 2022).

Based on the reviewed descriptions of methods, the proposed methods were often elaborately adapted and their parameters optimized for the same data or at least data from other participants following the exact same experiment design, on which all pipelines were later evaluated and compared. This practice is exposed to the so-called researcher bias. In contrast, other pipelines that are compared with the proposed method are often not or hardly adapted for the present use case, which can further introduce a bias toward the proposed method. As a result, the mean normalized rank of all proposed methods is 0.4088, while that of all other methods is 0.6324 (lower is better). Considering the fact that the latter paper does not necessarily compare their work on existing successful pipelines, the better score for proposed methods might not be caused by an improvement of methods but rather by the advantage of proposed methods due to this researcher bias.

It was shown by Tost et al. (2021) and Fló et al. (2022) that a parallelization of two or more streams within a pipeline with different preprocessing could increase the robustness and performance. Some authors have investigated the incorporation of accelerometer data into the pipeline and found a phase shift between the acceleration of the head. This is due to the neck muscles compensating head movements in full body movements and therefore the myogenic artifacts in EEG are delayed compared to the head acceleration (Kline et al., 2015; Nathan and Contreras-Vidal, 2016). Classical decomposition methods like ICA and ASR are not suited to model this phase delay, but specialized kernel methods such as CNN are.

#### The well known limitations of ICA

Most reviewed work focuses on approaches that optimize model parameters based on criteria measuring the independence of components, such as ICA. These methods come with strict assumptions that should be discussed in detail. Jung et al. (2001) explained four inherent assumptions of ICA clearly:

- The signal of each source summarizes linearly in the EEG channels.
- Spatial projections of components are fixed in time and conditions.
- Temporal independence of the components is given.
- The source signals have to be distributed non-Gaussian (i.e., a kurtosis not close to zero).

Even though some authors note that some assumptions do not or only hold to a certain degree, most agree that ICA is still very effective and stable for EEG data (Jung et al., 2001; Iriarte et al., 2003; Tamburro et al., 2018). Though, some issues are noted, for example varying tissue density in the brain affecting the first assumption (linear summary of each source signal) and some myogenic activities occurring regularly after the mental response affecting the third assumption “temporal component independence” (Kline et al., 2015; Nathan and Contreras-Vidal, 2016). However, it is out of the scope of this literature review to show whether the assumptions of ICA can be met for BCI systems.

A rather practical problem is discussed frequently in the reviewed papers: ICA is constrained in the number of independent components that it can extract from a given signal (Jung et al., 2001; Iriarte et al., 2003; Chen et al., 2014; Delisle-Rodriguez et al., 2017; Oliveira et al., 2017; Li et al., 2018; Sebek et al., 2018; Tamburro et al., 2018; Mur et al., 2019; Beach et al., 2021; Saba-Sadiya et al., 2021). The upper bound is given by the number of EEG channels, as a quadratic demixing matrix is used to reconstruct the source signals (Sebek et al., 2018). Therefore, a BCI system using the ICA method requires a high number of EEG channels which might mitigate the comfort of wearing it. In particular, for more frequent and more heterogeneous muscle activity, an increasing number of independent components are occupied to extract these components, thus reducing the IC containing useful mental signals (Chen et al., 2014; Anders et al., 2020; Kumaravel et al., 2022). This questions the validity of the ICA method for BCI systems in real-world applications.

Solutions to this limitation might be applying single-channel decomposition methods, as implemented by multiple authors (Chen et al., 2014; Roy et al., 2017; Liu et al., 2019; Mucarquer et al., 2020; Saini et al., 2020; Dai et al., 2021), who used the EMD method in advance of ICA and CCA. Another solution could be the usage of the Moore-Penrose Pseudoinverse to address the problem of the matrix inversion. This approach is used to calculate the inverse of a non-quadratic and therefore not fully ranked matrix, which can be used to reconstruct the brain signals from the EEG channel signals.

#### Research gaps for system development

Traditionally, BCI experiments are conducted with careful paradigms to avoid motion artifact contamination, rather than correcting those artifacts from the signal. By using combined hardware and signal processing for motion artifact removal, Nordin et al. (2019) found it is possible to identify human brain activity even when humans stepped over obstacles during walking and running. According to Nordin's research (Nordin et al., 2019), there were over 2,800 studies on human EEG published in 2017, yet >1% were on mobile subjects.

The majority of reviewed literature focuses on offline studies with not fully automated pipelines or on detecting and rejecting artifacts without any correction of the original EEG signal. More research in the fully automated correction of motion artifact contaminated EEG signals is needed (Yong et al., 2012; Zhang et al., 2015; Anders et al., 2020).

In the reviewed literature, we found no research that investigated changes in alpha and beta power on motion tasks other than treadmill walking. Building real BCI systems for real domains requires more research from other activities and domains such as collaborative robotics, sports, and working environments. In the case of treadmill walking, Nordin et al. (2020) found that the alpha/beta power increased during contra-lateral limb single support and push-off, and decreased during swing at each gait speed (Seeber et al., 2014; Wagner et al., 2014). At faster walking speeds spectral power fluctuations had limited duration and bandwidth, along with reduced alpha and beta power across the gait cycle, after muscle artifact removal. According to the authors, further research is needed that investigates the effects on the somatosensory cortex and motor cortex at the same time and the spectral power for tasks that involve greater amounts of sensory feedback built into motor execution. Reduced sensorimotor spectral power could be an indicator of greater cortical resources attuned to sensory feedback at faster locomotion speeds.

For successful practical implementation of real online BCI systems, more studies are needed to solve the decoding performance problem from incomplete EEG signals, rather than fully rejecting heavily contaminated segments (which is a problem for long-term learning strategies too). Consecutive and smooth recognition of BCI systems is needed for online and long-term applications. This requires that the BCI system can continuously decode brain signals without any interruption. If entire EEG segments are discarded due to extreme artifacts or data loss, the BCI system cannot obtain the decoding results during the corresponding time slice. Hence, it is very important to decode incomplete EEG in case of extreme artifacts and data loss (Chu et al., 2018).

#### Lack of advanced machine learning approaches

Authors of the reviewed literature reported that classifiers based on features of data decompositions (temporal, spectral, spatial) show poor generalizability for re-usage in other intended experimental setups (Lawhern et al., 2012; Frølich and Dowding, 2018; Tamburro et al., 2018). There is a need for more advanced time series models and the validation of their transferability to different recordings, hardware, mental strategies, sessions, subjects, and sensor layouts.

It has to be noted that participant metadata was never incorporated into models. Classificators could profit from additional information about the participant and setup such as the age, sex, EEG electrode type, and BCI paradigm. For example, the brain signals differ a lot for, e.g., children.

Crowdsourcing platforms for labeling artifacts such as ALICE (http://alice.adase.org/) and ICLabel (https://labeling.ucsd.edu/tutorial) emerge, that are suited for robust benchmarking newly proposed pipelines on a large dataset. However, both of them focus on the classification of artifacts based on independent components originating from an ICA. This enables the benchmarking of methods such as ADJUST, adjusted-ADJUST, RELICA, IClabel, FASTER, MARA, SASICA, and BeamICA, but are limited to the analysis of independent components. Crowd-sourcing platforms should be extended to classify artifacts based on the raw EEG time series instead of already decomposed signals. Moreover, indicating the probabilities of artifact labels based on multiple expert judgments could improve the model training, especially for rare artifacts (Soghoyan et al., 2021). It is also noteworthy, that the labels assigned by various experts were found to be more different than those of any IC-classification algorithm (Delorme, 2022). The crowdsourcing platforms should therefore also be used to discuss different opinions of experts such that automated algorithms can be trained on consistent and reliable labels.

Several authors have noted the lack of deep learning strategies applied to the detection, removal, and correction of artifacts in EEG signals (Val-Calvo et al., 2019; Nahmias and Kontson, 2021; Saba-Sadiya et al., 2021). While most existing work focuses on approaches that optimize model parameters based on criteria measuring the independence of components (ICA, PCA, CCA, etc.), recent advances in deep learning offer promising methods, which EEG pipelines could benefit from.

For instance, a two-layered perceptron (MLP with 2 layers) can implement the internal logic of an ICA. By adding more layers and non-linear mappings between them, the MLP can additionally correct high amplitude artifacts and select components useful for decreasing an appropriately chosen error criterion during training. A convolutional layer (CNN) can model time dependence between channels and generate time-dependent features from the input. More advanced deep learning methods such as variational autoencoder (VAE) and generative adversarial networks (GAN) can reconstruct artifact-free EEG signals from the original data. While these methods are already used in state-of-the-art active noise canceling systems, they have been applied to EEG data in only one single reviewed publication (Saba-Sadiya et al., 2021).

Furthermore, recent advances in training paradigms such as curriculum learning and self-supervised learning show promising results for deep learning models on complex data. Curriculum learning, for example, presents training examples to the model in a curriculum, starting with easy examples and gradually increasing the difficulty over time, allowing the model to learn gradually from simpler to more complex examples (Bengio et al., 2009). Self-supervised learning, on the other hand, allows a model to learn from input data itself without the need for explicit human annotation, enabling the training of large amounts of data with only a small fraction of labeled examples (Tian et al., 2020). None of these training concepts were found in the investigated publications.

It is important to note that incorporating advanced deep learning methods and paradigms requires a high degree of multidisciplinarity among the experts conducting the research. A deep learning expert must have a solid foundation in theory and practice to deal with complex time series data, as well as an understanding of the domain of EEG or electrophysiological data. Only then deep learning can be effectively applied to address the challenges posed by EEG artifacts.

## Conclusion

In conclusion, this systematic literature review compared and analyzed a large body of research on motion artifact reduction in brain-computer interface experiments using the PRISMA method. We aimed to create a comprehensive lookup table for the community to facilitate comparison and analysis of existing architectures and methods and to provide inspiration for further research.

Our findings revealed a potential publication bias toward newly introduced pipelines/methods over existing ones and the need for additional neutral method comparison studies by independent researchers. We also identified a gap in studies addressing the ground truth problem beyond measuring activity with additional sensors, such as separating individual muscle contributions from general muscle contamination or true brain components from others using creative hardware setups.

Furthermore, we observed limitations of ICA and similar methods for further exploitation in the field and recommended investigating advanced machine learning concepts in addition or comparison with traditional approaches. Customization and fine-tuning of BCI systems toward individual participants and users using machine learning could hold great potential for advancing the field.

We also noted that sample sizes of BCI studies are often small, and comparing data across multiple studies and datasets is challenging due to variations in paradigms, participant introductions, recording environments, hardware setups, and preprocessing steps. Addressing these challenges by incorporating crowdsourcing platforms and achieving a better understanding of motion artifacts by encouraging discussions between experts on them is crucial for the advancement of BCI systems that are usable in daily life settings.

In summary, this literature review highlights the need for further research in motion artifact reduction in BCI experiments, including neutral method comparison studies, addressing the ground truth problem, exploring advanced machine learning concepts, and overcoming challenges in sample sizes and data comparison. These findings provide valuable insights for researchers and practitioners in the field of BCI, and can guide future research directions for improving the effectiveness of motion artifact reduction methods in BCI experiments.

## Author contributions

MS-T: conceptualization, methodology, literature review, and writing. CS: methodology for literature comparison, literature review, analysis, and writing. GM-P: supervision, reviewing, and editing. All authors contributed to the article and approved the submitted version.
